# Effects of agricultural biodiversity and seasonal rain on dietary adequacy and household food security in rural areas of Kenya

**DOI:** 10.1186/s12889-015-1755-9

**Published:** 2015-04-25

**Authors:** Florence K M’Kaibi, Nelia P Steyn, Sophie Ochola, Lisanne Du Plessis

**Affiliations:** Kenya Technical Teachers College, Nairobi, Kenya; Division of Human Nutrition, Department of Human Biology, University of Cape Town, Anzio Road, Cape Town, South Africa; Department of Food, Nutrition and Dietetics, Kenyatta University, Nairobi, Kenya; Division of Human Nutrition, Stellenbosch University, Cape Town, South Africa; Division of Human Nutrition, Department of Human Biology, Faculty of Health Sciences, UCT Medical campus, Anzio Road, Anatomy Building, Floor 2, Room 2.04, Observatory 7925 Cape Town, South Africa

**Keywords:** Dietary intake, Dietary adequacy, Biodiversity, Household food security, Kenya

## Abstract

**Background:**

Kenya has a high prevalence of underweight and stunting in children. It is believed that both agricultural biodiversity and seasonal rainfall influences household food security and dietary intake. In the present study we aimed to study the effects of agricultural biodiversity and seasonal rains on dietary adequacy and household food security of preschool Kenyan children, and to identify significant relationships between these variables.

**Methods:**

Two cross-sectional studies were undertaken in resource-poor households in rural Kenya approximately 6 months apart. Interviews were done with mothers/caregivers to collect data from randomly selected households (N = 525). A repeated 24-hour recall was used to calculate dietary intake in each phase while household food security was measured using the Household Food Insecurity Access Scale (HFIAS). A nutrient adequacy ratio (NAR) was calculated for each nutrient as the percent of the nutrient meeting the recommended nutrient intake (RNI) for that nutrient. A mean adequacy ratio (MAR) was calculated as the mean of the NARs. Agricultural biodiversity was calculated for each household by counting the number of different crops and animals eaten either from domestic sources or from the wild.

**Results:**

Dietary intake was low with the majority of households not meeting the RNIs for many nutrients. However intake of energy (p < 0.001), protein (p < 0.01), iron (p < 0.01), zinc (p < 0.05), calcium (p < 0.05), and folate (p < 0.01) improved significantly from the dry to the rainy season. Household food security also increased significantly (p < 0.001) from the dry (13.1 SD 6.91) to the rainy season (10.9 SD 7.42). Agricultural biodiversity was low with a total of 26 items; 23 domesticated and 3 from the natural habitat. Agricultural biodiversity was positively and significantly related to all NARs (Spearman, p < 0.05) and MAR (Spearman, p < 0.001) indicating a significant positive relationship between agricultural biodiversity of the household with dietary adequacy of the child’s diet.

**Conclusion:**

Important significant relationships were found in this study: between agricultural biodiversity and dietary adequacy; between agricultural biodiversity and household food security and between dietary adequacy and household food security. Furthermore, the effect of seasonality on household food security and nutrient intake was illustrated.

## Background

Kenya is classified by the Food and Agricultural Organization (FAO) as a low-income-food deficit country [1]. It is among the one third of African countries whose food availability shows an average daily caloric availability below the recommended level of 2100 Kilocalories [2]. A recent economic review indicated that 51% of the population lack access to adequate food [3]. This inaccessibility to food is closely linked to poverty which stands at 46% [4]. The country has been facing serious food insecurity due to reduced cereal production, livestock diseases, rising food prices and poor rainfall. The food shortage situation was declared a national disaster at the beginning of January 2009 and May 2011 indicating that about 10 million persons were highly food insecure [5,6]. The most recent Democratic and Health Survey findings on child nutritional status showed that 16.1% of children aged below 5 years were underweight and 35.3% were stunted [7], indicative of poor household food security in a large proportion of the population.

Agricultural biodiversity helps to promote development and improves household food security [8]. There has, however, been a decrease in agricultural biodiversity in many developing countries, which has led to a reduction in the variety of animals reared for food and plants grown by households or picked in the wild [9]. This has led to a simplification and decrease in diversity of diets of a large number of people to a limited number of energy food sources that may not confer specific micronutrients, essential amino acids and essential fatty acids [10]. There is limited evidence of studies in sub-Saharan Africa linking agricultural biodiversity with household food security and nutritional status. In order to improve nutritional status it is therefore crucial to study the role of biodiversity as a factor which impacts on household food security [11].

In households with poor food security, low quality, monotonous diets are the norm. These diets generally constitute a large proportion of starchy foods which include cereals and tubers and are low in vegetables, fruits and animal protein [12,13]. The diets tend to be low in a number of micronutrients, and the micronutrients they contain are often not bio-available, thus resulting in deficiencies [13]. The risk of such deficiencies is high, particularly in children under the age of five years.

Undernutrition, including micronutrient deficiencies in early childhood may lead to a number of cognitive and physical deficits and may cause similar deficits in future generations as malnourished girls, particularly those with stunted growth, who become mothers, are at increased risk of giving birth to low birth weight infants [14]. The effects of undernutrition on human performance, health and survival have been the subject of extensive research for several decades [15]. Studies show that undernutrition affects physical growth, morbidity, mortality, cognitive development, reproduction, and physical work capacity [15]. Evidence from research carried out in developed countries show that dietary diversity is strongly associated with nutrient adequacy. A number of researchers from developing countries have also shown this association [16-21].

A study in Kenya by Ekesa et al. [22], showed a strong relationship between agricultural biodiversity and dietary diversity. The findings showed that almost 50% of changes in dietary intake of preschool children were due to changes in agricultural biodiversity. This implies that improving biodiversity can improve dietary diversity, which in turn can lead to an improvement in nutritional status [22]. In the present study we aimed to study the effects of agricultural biodiversity on dietary adequacy and household food security of preschool Kenyan children 24–59 months old, and to identify significant relationships between these variables.

## Methods

### Sample

Based on an effect size of 0.4 with 90% power and a significance level of 5%, a sample of 500 respondents (250 in each area) was required. The latter was based on the current national statistics for stunting (35%) in under-five children. The areas studied were resource-poor households in two rural districts of Meru in Eastern Province, Kenya namely: Akithii and Uringu. Uringu has a better rainfall and geographic resources compared to Akithii however in other respects the districts are similar being about 25 km apart. The households were randomly sampled by means of table of random numbers. A slight oversampling was done resulting in a total of 261 participants from Uringu division and 264 from Akithii division (N = 525). Two cross-sectional studies were undertaken, approximately 6 months apart.

The first phase of the study was conducted during the dry season and the second phase after the rainy season. The dry season took place when the food stores were low. October-November is a period when residents are most hungry since it is before the rains came. The rainy season took place when the food stores were normally good in this area since it was after the harvest of the short rainy period. The repeated surveys were not at the same households but households were randomly selected during both phases of the study. Interviews were conducted by trained nutrition graduates from Kenyatta University with mothers/caregivers of children aged 24–59 months.

### Data collection

#### Socio-economic and demographic questionnaire

The socio-demographic part of the questionnaire elicited information on the socio-economic status of the household; particularly questions on household assets. The latter have an influence on the economic status of the household which could in turn influence household food security.

### Dietary intake and adequacy

Dietary intake of each child was measured using a repeated 24-hour recall [23] with the mother/care giver of the index child in the household. Several days lapsed between the repeated interviews. Two 24-hour recalls were conducted in the dry season and two in the rainy season. The mother was asked to report all the food and drinks consumed by the subject during the previous 24 hours, starting with the first meal of the day and ending with the foods eaten last before bed time. In order to assist her with recall she was taken through the child’s activities of the day. In order to determine food portion sizes the interviewer used life-size photographs of food portions [24]. Standard size household utensils such as spoons, cups and mugs were also used to assist in clarifying the amounts of foods and liquids consumed. After the interviews the dietary data was coded and analyzed using food composition tables [25].

In order to determine the nutrient adequacy of the diet the nutrient adequacy ratio (NAR %) was calculated for each of 12 nutrients and energy, namely: vitamins A, B6, B12, C, B1, B2, niacin, folate; minerals- calcium, iron, and zinc and protein and energy. NAR% was calculated as being the % of the nutrient consumed, divided by the recommended nutrient intake (RNI) using the FAO/WHO recommended nutrient, energy and protein intakes [26-28]. The FAO/WHO RNIs were used because they are regarded as being more suitable for developing countries mainly due to the fact that they take into consideration the bioavailability of iron and zinc. The RNI = EAR + 2SD_EAR_ [26]. For iron and zinc the category of moderate bioavailability was used in this study. Each child was analyzed within their own age nutrient category when doing the dietary data analysis. This meant that cut-off points for the individual age groups were used. Once the NARs were calculated the mean adequacy ratio (MAR) of the diet was determined by the sum of each NAR divided by the number of nutrients. For both NAR and MAR 100% is the ideal since it means that the intake is the same as the requirement.

### Agricultural biodiversity

Penafiel et al. [29], described the assessment of local biodiversity as listing the local edible plants and animals included in the diet of the population. The Food and Agriculture Organization (FAO) [30], proposed developing an inventory of food biodiversity available from key informants and interviews or focus group discussions.

In the present study the researchers constructed a questionnaire using guidelines from FAO [30]; for developing indicators for monitoring agricultural biodiversity and also from a previous study undertaken in Kenya [22]. This questionnaire was pretested to improve its validity. In-depth interviews were held with key informants (village elders) and 4 focus group discussions with 8–12 participants were held with those deemed to have knowledge of local foods, to corroborate data obtained by questionnaire.

Agricultural biodiversity was measured by determining the variety of food plants grown, animals reared for food and food items obtained from natural habitats in the past year. A list of all food items grown, all animals reared and hunted, and other food items obtained from natural habitats through gathering or trapping was determined for each household by means of a short questionnaire which asked the participant to list all the food items utilized over the past year (dry and rainy seasons). Food items purchased from markets or towns were not included in the agricultural biodiversity score.

A score of biodiversity was calculated for each household according to which indigenous and cultivated food items were used at any time by the household over a period comprising the past year. The maximum found was 26. Each household’s biodiversity score was then correlated with the individual nutrient adequacy ratios from the repeated 24 hour recalls of the child participant in that household.

### The Household Food Insecure Access Scale (HFIAS)

Food security was assessed by means of the HFIAS developed by Coates et al. [31]. The HFIAS is internationally used and is regarded as being a valid instrument for this purpose. This assessment tool is based on the principle that the experience of food insecurity causes predictable reactions and responses that can be captured and quantified through a survey and summarized in a scale. The nine-item scale uses a four-week recall period and was constructed to capture three larger dimensions of household food insecurity: anxiety and uncertainty about household food access: insufficient quality and insufficient food intake and its physical consequences or hunger [31].

The information generated by the HFIAS was used to assess the prevalence of household food insecurity and to detect changes in the household food security situation of the population during the two seasons, namely the dry season and rainy season (after harvest season). Since the study period included both seasons, HFIAS generic questions were adapted and translated to ensure that questions were understood in their cultural context. The first phase of the study took place when the food stores were low. October-November is a period when respondents are most hungry since it is before the rains come. The second phase of the study, took place when the food stores were normally good in this area since it was after the harvest of the short rainy period.

The HFIAS was used to determine the prevalence of household food insecurity. The HFIAS is a continuous measure of the degree of food insecurity in the household in the past four weeks (30 days). First, a HFIAS score variable was calculated for each household by summing the codes of each frequency-of-occurrence question. The maximum score for a household is 27. The higher the score, the more food insecurity (lower access) the household experienced. The lower the score, the less food insecurity a household experienced [31]. In order to report household food insecurity prevalence (HFIAP) [31], the HFIAP indicator was used to categorize households into four levels of household food insecurity: i) food secure (0–1) ii) mildly food insecure, (2–9) iii) moderately food insecure (10–14) and iv) severely food insecure (15+).

### Ethics

The study was approved by the Committee for Human Research, Faculty of Medicine and Health Sciences, Stellenbosch University (ethics reference No. N11/02/037). Each participant was required to sign a consent form after the purpose of the study had been explained to them. Thumb prints were used for participants who could not write. The researcher also obtained permission to conduct the research from the National Commission of Science, Technology and Innovations of Kenya.

### Data analysis

The entry of the raw data was done using Microsoft Access 2003 and exported to MS Excel 2003. Data cleaning was done before the data was transported to the data analysis packages. STATISTICA version 9 (StatSoft Inc. (2009) STATISTICA (data analysis software system) (www.statsoft.com), Statistical Package for Social Sciences (SPSS Version 11.5) were used to analyze the data. Food finder 3, [25], was used to analyze the dietary data that was collected using the 24-hour recall. This is a software product developed by the Medial Research Council of South Africa [25]. Kenyan foods were added to the database from previous studies.

## Results

Forty-one percent of mothers/caretakers were casual laborers, 19.5% were petty traders, 5.4% were unemployed, 4.5% were self-employed and 1.2% were wage earners (data not shown). The majority (84.6%) of mothers/care givers had a primary level of education, 5.0% had some secondary education, 4.4% had completed secondary education and 5.0% had no formal education. Ninety-six percent of the respondents owned land which was under food production and all (100%) had a food or grain store in their homes. All (100%) the respondents were small scale farmers. The mean acreage of land under food production for both divisions was 1.4 ± 1.1. There was a significant difference in the size of farms under food production between Akithii and Uringu. [Akithii 1.5 ± 1.04 hectares, Uringu 1.2 ± 1.00 hectares (p < 0.001)]. Respondents from Akithii had relatively larger farms under food production compared to those in Uringu. Overall, in both areas participants owned their own homes (99.1%) (Table 1). Other assets owned by a substantial number of households were sofa sets, vegetable gardens and fruit trees. Significant differences between the two divisions were found in the ownership of radios (p = 0.019), sofa sets (p = 0.002), vegetable gardens (p = 0.015) and fruit trees (p < 0.0001). More residents of Uringu had vegetable gardens and fruit trees while a greater number of residents in Akithii had bicycles, sofa sets and cell phones.

**Table 1:** **Assets owned by families in the two study areas**

***Household assets***	***Akithii N = 261***		***Uringu N = 264***		***Total for both divisions***		***Chi-square p-values***
	**N**	**%**	**N**	**%**	**N**	**%**	
Own home	261	99.6	258	98.5	519	99.1	Χ = 1.829; p = 0.176
Television set	34	13	39	14.9	73	14.0	Χ = 0.421; p = 0.517
Radio	156	59.5	181	69.1	337	64.3	Χ = 5.487; p = 0.019*
Vehicle	2	0.8	6	2.3	8	1.6	Χ = 2.047; p = 0.153
Bicycle	135	51.5	121	8.0	256	29.8	Χ = 0.017; p = 0.896
Wheelbarrow	23	8.8	25	9.5	48	9.2	Χ = 0.100; p = 0.751
Sofa set	134	51.1	100	38.2	234	44.7	Χ = 9.202; p = 0.002**
Cell phone	194	74.0	183	69.8	377	71.9	Χ = 1.204; p = 0.272
Vegetable garden	75	28.6	101	38.5	176	33.6	Χ = 5.940; p = 0.015*
Fruit trees	81	30.9	206	78.6	287	54.8	Χ = 119.689; Χ = p < 0.001***

*p <0.05; **p<0.01; ***p< 0.001.

A comparison of the mean nutrient intakes of macronutrients between the two seasons (1 = dry season; 2 = after rain) is displayed in Table 2. Significant increases in mean nutrient intakes were found between the two phases in both areas, namely: energy (p < 0.001); carbohydrate (p < 0.001); added sugar (p < 0.001); total protein (p < 0.01); vegetable protein (p < 0.001); saturated fat (p < 0.05); and fiber (p < 0.001). Mean intakes were generally greater in Uringu compared to Akithii. Mean energy and fiber intakes were lower than the RNIs.

**Table 2:** **Macronutrient intakes of 24–59 month old children derived from repeated 24-hour recalls in the dry and rainy seasons of the two areas studied**

	**Dry season**				**Rainy season**				**Both combined**				
**Nutrient**	**Akithii** ^**a**^		**Uringu** ^**a**^		**Akithii**		**Uringu**		**Akithii** ^**b**^		**Uringu** ^**b**^		**FAO RNI**
	**Mean**	**SD**	**Mean**	**SD**	**Mean**	**SD**	**Mean**	**SD**	**Mean**	**SD**	**Mean**	**SD**	
Energy-(kJ)	3392	2069	3684	1599	3808***	1914	4149***	1673	3599	2002	3908**	1650	4276-5656
Carbohydrate (g)	127	67	134	55	149***	74	153***	60	138	71	143	58	-
Added sugar (g)	5.2	15.76	6.0	14.43	7.8**	14.54	12.3**	24.12	6.5	15.21	9.1*	19.93	-
Total protein (g)	20.6	12.33	22.3	11.98	23.9**	13.10	25.5**	11.78	22.2	12.81	23.8*	11.98	14-22.2
Animal protein (g)	1.4	3.11	2.1*	3.47	1.6	2.40	2.7*	4.35	1.5	2.78	2.4***	10.37	-
Vegetable protein (g)	19.1	11.51	18.7	10.47	21.9**	12.33	22.5***	9.92	20.5	11.99	20.5	10.37	-
Total fat (g)	17.5	22.95	21.1	17.86	15.9	15.51	22.1	18.39	16.7	19.60	21.6***	18.10	-
Poly-unsaturated fat (g)	4.9	5.90	6.6	6.37	4.1	4.14	6.2	6.40	4.5	5.11	6.4***	6.03	-
Saturated fat (g)	4.1	5.45	4.9	4.57	5.7*	9.36	5.7*	5.64	4.9	7.69	5.3	5.53	-
Fiber (g)	13.7	7.68	15.7	7.39	16.9***	9.83	17.8***	7.97	15.3	8.95	16.7***	7.74	19-25

^a^Significant difference between mean values using t-test: *p < 0.05; **p < 0.01; ***p < 0.001 between the dry and rainy seasons; ^b^between the two areas; RNI = recommended nutrient intakes.

A comparison of the mean micronutrient intakes between the two phases was done (Table 3). Mean intakes of calcium, zinc, vitamin A, riboflavin and niacin were below the RNIs. With regard to the two seasons it should be noted that there were significant improvements in certain micronutrients in the rainy season in both areas, namely calcium (p < 0.05); iron (p < 0.01); zinc (p < 0.0.05) and folate (p < 0.01). However the mean intake of vitamin A decreased in both areas.

**Table 3:** **Micronutrient intakes of 24–59 month old children derived from repeated 24-hour recalls in the dry and rainy seasons of the two areas studied**

	***Dry season***				***Rainy season***				***Combined seasons***				
**Nutrient**	***Akithii***		***Uringu***		***Akithii***		***Uringu***		***Akithii***		***Uringu***		***FAO RNI***
	**Mean**	**SD**	**Mean**	**SD**	**Mean** ^**a**^	**SD**	**Mean** ^**a**^	**SD**	**Mean**	**SD**	**Mean** ^**b**^	**SD**	
Calcium (mg)	146	108	196	137	155*	134	200*	154	151	122	199***	145	500-600
Iron (mg)	4.6	2.76	5.4	2.79	5.5**	3.68	6.3**	3.52	5.1	3.27	5.9*	3.19	6.0
Zinc (mg)	2.6	1.51	3.1	1.81	3.1*	1.71	3.5*	1.89	2.9	1.63	3.3**	1.86	4.1-6.1
Vitamin A (ug)	581*	577	587*	532	288	399	398	517	435	517	496**	533	400-450
Vitamin C (mg)	38	38.6	62*	51.1	34	41.3	57	47.4	36	40	60***	49.3	30
Folate (ug)	187	227.4	213	184.3	258**	181.9	261**	150.8	222	208.8	236	170	160-200
Thiamin (mg)	0.5	0.56	0.6	0.46	0.6	0.36	0.6	0.30	0.6	0.47	0.6	0.38	0.5-0.6
Riboflavin (mg)	0.3	0.57	0.3	0.48	0.3	0.27	0.3	0.22	0.3	0.45	0.3*	0.38	0.5-0.6
Niacin (mg)	3.3	5.31	4.6	5.37	3.2	2.79	4.2	3.35	3.2	4.24	4.4***	4.51	6-8
Vitamin 6 (mg)	0.5	0.40	0.6	0.41	0.5	0.36	0.6	0.39	0.5	0.38	0.6***	0.41	0.5-0.6
Vitamin B12 (ug)	0.2	0.81	0.2	0.33	0.2	0.29	0.3	0.41	0.2	0.61	0.3***	0.37	0.9-1.2

^a^Significant difference between mean values using t-test: *p < 0.05; **p < 0.01; ***p < 0.001 between the dry and rainy seasons; ^b^between the two areas; RNI = recommended nutrient intakes.

The lowest NAR values were found for vitamin B12 and calcium (Table 4). Vitamin B12 values were less than 25% and calcium less than 40% of the requirement, respectively. Energy and protein NARs were all less than 50% of the RNI. The highest NARs of 70% and above were found for vitamin B6, C, thiamin, folate and iron. When combining the micronutrients to provide a MAR value, it was found to be 55.3% for Akithii and 66.8% for Uringu in the dry season and 56.3 and 67.4 in the rainy season, respectively; representing an improvement which was not significant. Comparison of the means of the two seasons showed significant improvements in energy (p < 0.001); protein (p = 0.001); folate (p < 0.001; zinc (P < 0.01); and iron (p < 0.01). Uringu consistently had higher means for all the NARs, which means that the children in Uringu had a higher MAR than those in Akithii, reflecting a diet of better quality.

**Table 4:** **Nutrient adequacy ratios of nutrients and mean adequacy ratio of the nutrients of 24–59 month old children derived from repeated 24-hour recalls in the dry and rainy seasons**

	***Dry season***				***Rainy season***			
**Nutrient**	***Akithii***		***Uringu***		***Akithii***		***Uringu***	
	**Mean**	**SD**	**Mean**	**SD**	**Mean**	**SD**	**Mean**	**SD**
	**NAR**		**NAR**		**NAR**		**NAR**	
Energy	36.2	21.10	39.5	16.1	40.9***	20.42	44.5***	17.48
Protein	41.5	21.62	44.4	19.77	48.7***	26.61	52.0***	24.02
Vitamin A	66.2***	39.83	73.5***	34.14	44.8	39.14	61.5	33.70
Vitamin B6	67.1	30.72	85.8	20.23	71.0	30.25	85.5	21.36
Vitamin B12	15.4	26.01	21.9	26.65	18.4	23.41	25.7	28.31
Vitamin C	66.4**	38.81	89.4*	24.35	55.0	41.60	81.9	31.94
Niacin	43.7	25.52	60.0	26.71	46.8	28.42	57.80	25.65
Riboflavin	45.0	26.84	54.7	23.92	50.4*	26.50	58.4	24.54
Thiamin	77.7	24.25	84.0	21.43	80.7	23.93	87.4	18.73
Folate	74.4	30.96	85.0	22.08	85.4***	23.71	92.3***	17.42
Iron	67.0	29.88	77.4	23.59	72.0*	29.50	83.0***	20.13
Calcium	28.1	20.73	36.8	22.25	29.2	23.60	36.5	22.93
Zinc	57.5	27.46	67.1	24.04	65.1**	27.62	71.8*	22.66
**MAR** ^**##**^	**55.3**	**23.65**	**66.8**	**17.19**	**56.3**	**23.23**	**67.4**	**17.76**

Significance of t-tests: *p < 0.05; **p < 0.01; ***p < 0.001 between the two seasons; ^##^MAR=Mean adequacy ratio.

Table 5 shows that the cereal group was consumed by nearly all children followed by the vitamin A-rich fruits and vegetables. The next group most commonly consumed was the dairy group. Consumption of meat and eggs were very low in both areas. The table further indicates that there is an increase in percentage children consuming certain groups in the rainy season in both areas. These are non-vitamin A rich fruits and vegetables (A = 13.6% to 23.1%; U = 47.7% to 52%); sugars and sweets (A = 26.7% to 45.3%; U = 28.4 to 56.5%; legumes (A only = 17.9% to 28.8%); and dairy (A only = 50.4% to 61.9%). In both areas the percent children consuming vitamin A rich fruit and vegetables decreased (A = 74.5 to 59.6%; U = 91.3% to 78%). The 10 most commonly consumed food items were maize meal, maize with beans, tea, kale, sugar, spinach and potatoes, tomatoes, boiled maize and chapattis in the dry season and tea, maize meal, maize and beans, milk, sugar, onions, mango, rice and beans, tomatoes and potatoes in the rainy season (not shown).

**Table 5:** **Percent of children consuming foods from different food groups in the dry and rainy seasons**

**Food group**	**Akithii**		**Uringu**		**Akithii**		**Uringu**	
	**Dry season**		**Dry season**		**Rainy season**		**Rainy season**	
	**%**	**SE**	**%**	**SE**	**%**	**SE**	**%**	**SE**
Cereals, roots and tubers	98.5	1.0	96.2	3.8	98.9	0.68	98.2	0.9
Vitamin A rich fruits & veg	74.5	4.2	91.3	2.8	59.6	0.5	78.0	0.07
Other fruits & vegetables	13.6	6.0	47.7	7.4	23.1	4.9	52.0	1.3
Sugars, syrup and sweets	26.7	2.1	28.4	3.0	45.3	0.9	56.5	1.4
Legumes & nuts	17.9	2.5	46.4	0.2	28.8	6.3	44.6	4.3
Meat, poultry, fish	1.7	0.01	2.1	0.4	0.6	0.2	2.2	1.7
Fats & oils	13.8	5.3	18.9	1.9	13.7	0.1	11.7	0.3
Dairy products	50.4	2.5	79.4	4.9	61.9	2.1	78.7	0.2
Eggs	0.4	0.003	1.1	0.2	0.7	0.2	0.9	0.01
Beverages*	0.8	0.3	0.9	0.03	1.0	0.3	1.5	0.4

*When doing the 24 hour recalls, black tea or coffee were not included as foods used in calculating nutrient intakes since they do not contain any macro- or micronutrients. However if milk was added to the tea or coffee, the milk portion was added to the dairy group. Hence the beverages referred to in the table are cold drinks.

**Figure 1:**
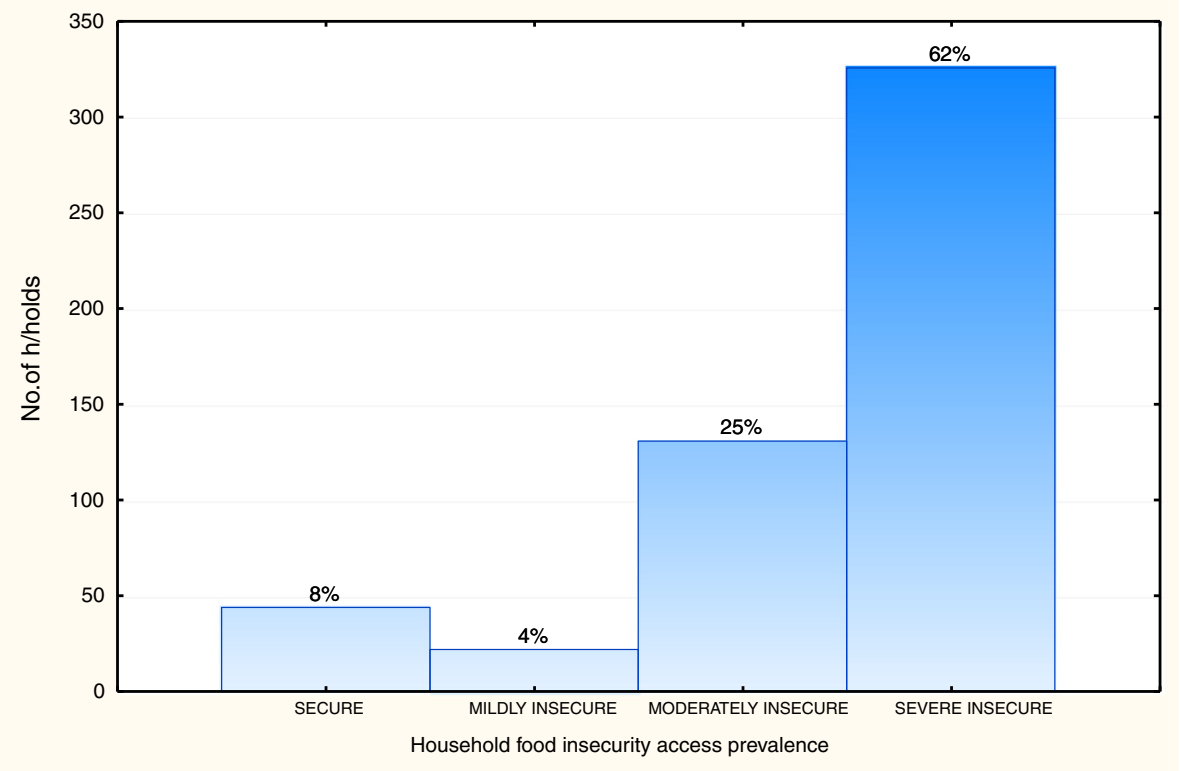
Household food insecurity access prevalence categories for the two divisions in the dry season.

**Figure 2:**
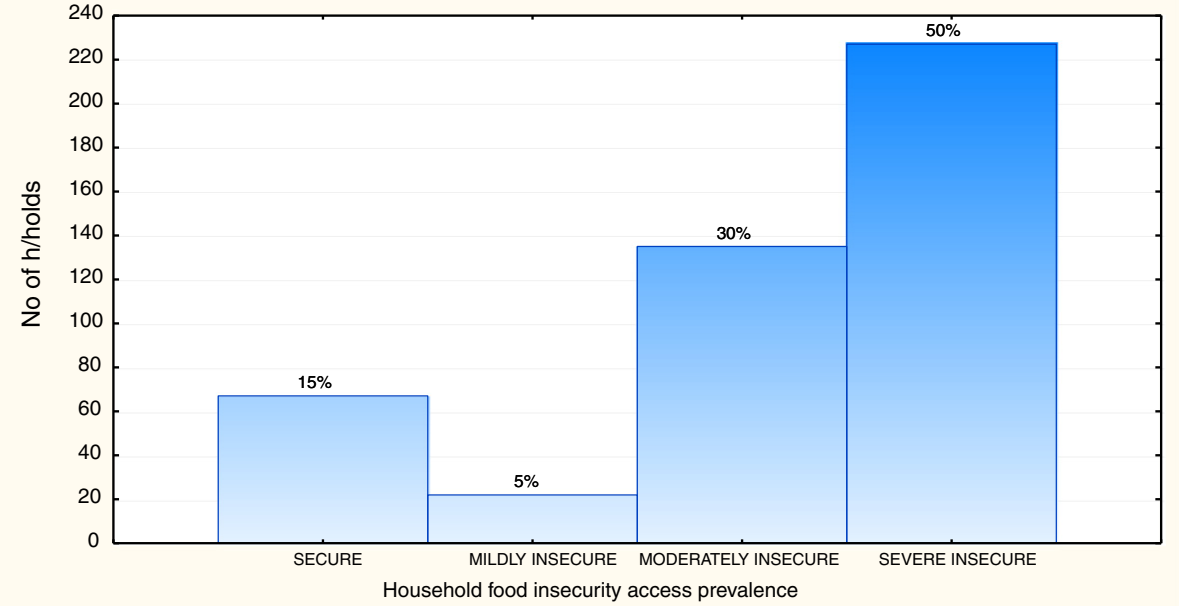
Household food insecurity access prevalence categories for the two divisions in the rainy season.

**Figure 3:**
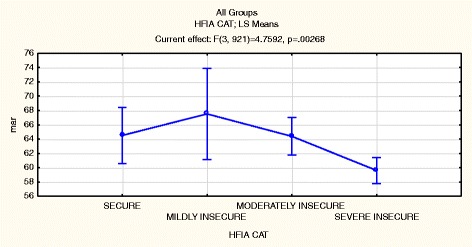
Comparing Household Food Insecurity Access categories (HFIACAT) with theMicronutrient Adequacy Ratio (MAR).

To assess whether the household food security situation was influenced by the change in seasonality, a comparison was done between the two seasons of data collection. Table 6 shows the HFIAS mean scores during the two seasons. For Akithii and both areas combined the scores are significantly higher during the dry season which is indicative of poorer household food security during this season. This is also illustrated in Figures 1 and 2 which show that the prevalence of severe food insecurity decreases during the rainy season. Figure 3 shows that households that were food secure were likely to have children with a higher MAR (p = 0.002). Households that were food secure and mildly food insecure had a higher MAR than those that were moderately and severely food insecure.

**Table 6:** **The household food security mean scores in the two areas studied during the dry and rainy seasons**

	**Akithii**	**Uringu**	**Combined**
	**Mean (SD)**	**Mean (SD)**	**Mean (SD)**
Dry season	16.2 (7.01)***	10.0 (6.90)	13.1 (6.91)***
Rainy season	12.5 (7.80)	9.3 (7.02)	10.9 (7.42)

***The two tailed p value <0.001 (t-test) indicates a significant difference between the means of the dry and rainy seasons. A lower score is indicative of better household food security.

The total number of different food items (agricultural biodiversity) in the two areas over the past year as reported by participants and focus groups are presented in Table 7. They include cultivated food items and those obtained from the natural habitat. The majority of items were cultivated (n = 23); with only three obtained from the wild. The latter being wild berries, Amaranthus blitum and antelope (deer).

**Table 7:** Total number of different food items (agricultural biodiversity) in the two areas over the past year as reported by participants* and focus groups

**Categories**	**Types of food items**		
	**Domesticated/cultivated**	**Natural habitat**	**Total number**
Animals	Goats, pigs, chicken, rabbit, sheep, ducks, cows	Antelopes	8
Cereals, pulses and roots	Maize, beans, sorghum, pigeon peas, cowpeas, millet, arrow roots	None	7
Nuts	Ground nuts, macadamia nuts	None	2
Fruits	Paw paws, avocadoes, bananas, oranges, mangoes	Wild berries	6
Vegetables	Kales and tomatoes	Amaranth sp Amaranthus blitum (terere)	3
Total biodiversity	23	3	26

*Mean agricultural biodiversity scores was 6.6 (SD 2.44) in Akhitii and 7.2 (SD 4.19) in Uringu.

Correlations between agricultural biodiversity scores of participants and their NARs are shown in Table 8. A significant relationship was found to exist between the agricultural biodiversity score with all the nutrients investigated in the study with the exception of energy. Since the correlations are positive it is noted that increased NAR (dietary adequacy) of the child is associated with an increased agricultural biodiversity score of the household.

**Table 8:** **Correlations between agricultural biodiversity score and nutrient adequacy ratios**

**Spearman rank order correlations**			
**Variables**	**Spearman - R**	**t(N-2)**	**p-value**
Biodiversity score & NAR Energy	0.085	1.905	0.057
Biodiversity score & NAR Protein	0.092	2.074	0.038*
Biodiversity score & NAR Iron	0.152	3.442	0.001***
Biodiversity score & NAR Zinc	0.130	2.921	0.003**
Biodiversity score & NAR Vit B12	0.118	2.663	0.007**
Biodiversity score & NAR Vitamin B6	0.193	4.381	p < 0.001***
Biodiversity score & NAR Vitamin C	0.176	4.003	p < 0.001***
Biodiversity score & NAR Folate	0.091	2.054	0.040*
Biodiversity score & NAR Riboflavin	0.184	4.172	p < 0.001***
Biodiversity score & MAR	0.194	4.405	p < 0.001***

NAR = nutrient adequacy ratio; MAR = mean adequacy ratio; Correlations are significant at *p <0.05; **p < 0.01; ***p < 0.001.

**Figure 4:**
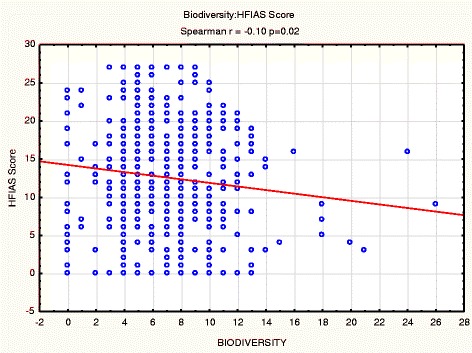
A correlation between the HFIAS score with household agricultural biodiversity scores.

A significant relationship was also found to exist between agricultural biodiversity and household food security (HFIAS) (Spearman, p = 0.02) (Figure 4). As the agricultural biodiversity score increased, the HFIAS score decreased, showing that an increase in agricultural biodiversity improved household food security.

## Discussion

In summary, results for dietary adequacy showed that children had poor intakes of energy, protein, fiber and numerous micronutrients. The low energy intake helps to explain the high degree of chronic malnutrition found in these children with stunting at 31.9-34.7% in Akithii and 26.23-28.2% in Uringu [32]. It is interesting to note that the children in Uringu were generally better off than those in Akithii in terms of dietary adequacy, food security and agricultural biodiversity. However, one of the most important outcomes of the study were the significant improvements in dietary adequacy and in household food security during the rainy season. In both areas there were significant increases in energy, carbohydrate, protein, saturated fat, sugar and fibre. Many micronutrients, including calcium, zinc, iron and folate also increased significantly in both areas in the rainy season. Vitamin A was the only micronutrient not to do so and this was likely due to the finding that the main vitamin A source (spinach and kale) was consumed in the dry season. Increases in the percentage children consuming certain food groups also showed an upward trend in non-vitamin A rich fruit and vegetables, sugar, legumes, and dairy products, in the rainy season.

Additionally, household food security as measured by the HFIAS also improved significantly during the rainy season, further emphasizing the importance of seasonal effects on households. These findings are similar to those of a study conducted in Mozambique that found that change in seasonality affected household food security as measured by the HFIAS [33]. Researchers who undertake dietary surveys in countries like Kenya need to be aware of the importance of including data from different seasons.

Kenya has been described as a country rich in agricultural biodiversity with an estimated 35,000 known species of animals, plants and micro-organisms [34]. The country's agricultural biodiversity is, however, under serious threat due to among others increasing deforestation, climate change, pollution and soil degradation [35]. The level of agricultural biodiversity (n = 26) and the mean scores (6.6 and 7.2, respectively) in the Eastern part of Kenya, the area of study, was found to be low and far less than the number described in an earlier study conducted in western Kenya which found 41 different species of food cultivated, animals reared and those foods from the natural habitat [22]. Our methodology was similar to the one used in this earlier study. However despite the lower figure, the present study showed a significant positive relationship between agricultural biodiversity and nutrient adequacy ratios (NARs) implying that as one increased so did the other.

A study by Frison [36] indicated that, in Kenya, rice, maize and wheat contribute about 60% of calories and proteins from plants. The magnitude of agricultural effort applied to the three principal crops has led to a decline in the production and consumption of more diverse grains. This concurs with the findings of the present study which revealed that the production of cereals such as indigenous millet and finger millet has declined and the number of foods which can be obtained from the natural habitat have been significantly reduced. This further corresponds with a study by John, [10] which indicated that cultivation of traditional foods like: millet, sorghum, cassava, sweet potatoes, traditional vegetables and indigenous wild fruits are now associated with being poor. This association results in changes in agricultural practices, which lead to disruption of dietary patterns and loss of dietary diversity. The 10 most common food items noted in this study did not include any indigenous foods mentioned above and comprised largely of maize, rice, potatoes and wheat as staple foods.

The relationship between agricultural biodiversity and dietary adequacy (in terms of NARs) was explored in order to quantify the relationship between dietary adequacy and agricultural biodiversity. Highly significant positive correlations were found between agricultural biodiversity and NARs of calcium, iron, zinc, vitamin A, B6, C, folate, riboflavin, protein and energy, indicating the very strong relationship between dietary adequacy and biodiversity. These findings are in agreement with those of other studies which showed a strong relationship between these variables [37,38]. The significance of this finding is emphasized by realizing the importance of maintaining or improving biodiversity in populations which are dependent on the land for food [38-40].

Recognition of the value of maintaining and using agricultural biodiversity is not new [38-40]. A significant relationship was found to exist between agricultural biodiversity and food security in this study. As the agricultural biodiversity score increased, the HFIAS score decreased showing that an increase in agricultural biodiversity improved household food security (access). There is limited evidence in SSA of studies linking agricultural biodiversity with household food security and nutritional status. This study showed a significant relationship between agricultural biodiversity and household food security concurring with the recommendation by Frison [11] that it is crucial to study the role of biodiversity as a factor which impacts on household food security. Kenya plans to reduce food insecurity by 30% by 2015 [41]. Maintaining and improving agricultural biodiversity should therefore form part of the interventions to enable the achievement of this target, especially in rural areas.

To assess whether household food security was influenced by the change in seasonality, a comparison was done between the dry season and the rainy seasons. There were significant differences between results of the two seasons; with the dry season showing relatively higher levels of food insecurity compared to the rainy season.

Certain limitations of the study need to be noted. Firstly, the two areas studied were not as similar regarding their agricultural and physical resources despite the fact that they were fairly close in physical proximity. Secondly, when evaluating agricultural biodiversity we only examined food items which were cultivated or obtained from the wild. We did not determine the extent to which foods were purchased from stores and markets.

## Conclusion

The dietary intakes of macronutrients and micronutrients were low in this study with most of the preschool children not meeting the recommended nutrient intakes. The following important significant relationships were found in this study: between agricultural biodiversity and dietary adequacy; between agricultural biodiversity and household food security and between dietary adequacy and household food security. Furthermore, the effect of seasonality on household food security and dietary intake of the children was illustrated.
